# A Gender-Based Descriptive Analysis of the Canadian Network Undertaking Against Hepatitis C (CANUHC) Cohort From 2015–2023

**DOI:** 10.1093/ofid/ofag356

**Published:** 2026-06-06

**Authors:** Mia J Biondi, Haris Imsirovic, Jordan J Feld, Hongqun Liu, Alexa Keeshan, Samuel S Lee, Curtis L Cooper

**Affiliations:** York University, Toronto, Ontario, Canada; Viral Hepatitis Care Network, University Health Network, Toronto, Ontario, Canada; Ottawa Hospital Research Institute, Ottawa, Ontario, Canada; Viral Hepatitis Care Network, University Health Network, Toronto, Ontario, Canada; University of Calgary, Calgary, Alberta, Canada; Ottawa Hospital Research Institute, Ottawa, Ontario, Canada; University of Calgary, Calgary, Alberta, Canada; Ottawa Hospital Research Institute, Ottawa, Ontario, Canada; University of Ottawa, Ottawa, Ontario, Canada

**Keywords:** comprehensive care, gender-diverse persons, hepatitis C virus, women

## Abstract

Understanding the impact of gender has been identified a priority focus in regional and global efforts to eliminate hepatitis C virus (HCV) as a public health threat. Gender affects health and wellness across the lifespan, and women and gender-diverse peoples face increasing structural barriers, leading to a higher risk of HCV acquisition and decreasing the ability to seek care and treatment. The objective of this study was to describe a Canadian cohort of individuals engaged in HCV care self-identified as cis or trans women or trans men. In this cohort of cis women and trans persons, fibrosis scores were low, but specific groups were more likely to have compensated/decompensated cirrhosis, highlighting the need for earlier case-finding and treatment. In addition, opioid agonist therapy (OAT) uptake was poor, an observation that may support co-localizing HCV treatment with OAT initiation to prevent drug use relapse and reinfection.

Cis women and gender-diverse peoples face systemic and social inequities that impact their exposure to hepatitis C virus (HCV) prevention and treatment. These inequities are even more significant for those involved in sex work or sexual exploitation, those who use drugs, newcomers and immigrants, and those with experience in corrections, among other groups. There are an estimated 15 000 000 women of childbearing potential living with HCV globally [1], and there has been a 20% increase among women in high-income countries in the last 25 years [2]. Among trans and nonbinary individuals, gaps in clinical outcomes have been identified in HCV care and treatment [3, 4].

Gender-based approaches to HCV elimination have been prioritized by the World Health Organization and others, yet few studies have described disaggregated data for cis women and trans persons [5, 6]. Using a large prospective cohort from Canada, the objective of this study was to describe those who self-identified as women or trans persons, including detailed demographics, HCV-related care, and clinical outcomes.

## METHODS

We conducted a retrospective analysis of a prospectively collected cross-sectional cohort study using a large data set from Canada, the Canadian Network Undertaking Against Hepatitis C (CANUHC) cohort. CANUHC contains prospectively collected demographic information and HCV treatment information from 17 Canadian-based, publicly funded HCV clinic sites from January 2015 to May 2023. This cohort included HCV patients with biological sex identified as female, as well as patients of male sex who identified as either trans or female gender. Individuals with missing information on sex but gender-identified as female were also included. Demographic information, baseline clinical data, and treatment outcomes were reported (overall and stratified). The proportions that were cirrhotic and noncirrhotic, or reinfected and nonreinfected were compared among demographic subgroups using the chi-square test. No adjustments were made for multiple comparisons. Clinicians completed a standardized intake form to identify comorbidities, HIV-1, hepatitis B virus (HBV), complete blood count (CBC), alanine transaminase (ALT), aspartate aminotransferase (AST) , HCV viral load, genotype, fibrosis stage, past exposure and treatment, and current treatment. Patients were followed from their index assessment until sustained virologic response (SVR)/HCV cure, death, or loss to follow-up if they initiated direct-acting antiviral (DAA) treatment.

### Patient Consent

The patient's written consent was obtained. The use of these data was approved by Ottawa Hospital Research Institute (20170580-01H).

## RESULTS

Within CANUHC, 1142 cis women and 25 trans persons were identified. Demographics, life experiences, comorbidities, liver disease assessment, and treatment are reported (Table 1). Age ranged from 17 to 89 years, and the mean age (SD) was 48.8 (14.0) years. Seventeen (1.5%) patients self-identified as trans women, and 8 (0.7%) identified as trans men. Most patients in the cohort were white (63.5%). Of the cohort, 38.6% had a college or university degree, 29.7% were employed, and 10.6% had housing insecurity; 38.1% were either married or common law, and 73.0% had children. Most individuals identified as heterosexual (91.7%) and did not engage in high-risk sexual activities (71.1%). A high proportion had a history of incarceration (28.1%). Thirty-five percent of the cohort consumed alcohol, and 3.9% used hazardous levels of alcohol (>3 drinks per day). Most of the participants had used recreational drugs in the past (70.6%). While those with a history of injection drug use comprised 62.9%, 75.0% of the cohort was not on OAT. Comorbidities included diabetes (6.7%), psychiatric diagnosis (36.5%), chronic renal disease (1.4%), HIV (5.6%), and HBV (2.2%) coinfection.

**Table 1.** Baseline Demographic and Clinical Characteristics of Cis Women and Trans Persons in CANUHC (n = 1167)

Characteristic	Value
Province, No. (%)	
Alberta	294 (25.2)
British Columbia	277 (23.7)
New Brunswick	91 (7.8)
Nova Scotia	78 (6.7)
Ontario	278 (23.8)
Quebec	30 (2.6)
Saskatchewan	119 (10.2)
Clinic setting, No. (%)	
Academic	838 (72.1)
Community	324 (27.9)
Age, mean (SD), y	48.8 (14.0)
Gender (self-identified), No. (%)	
Cis women	1142 (97.9)
Trans women	17 (1.5)
Trans men	8 (0.7)
Race/ethnicity (self-identified), No. (%)	
Asian	106 (9.3)
Black	29 (2.5)
Indigenous	240 (21.0)
White	726 (63.5)
Other	42 (3.7)
Immigrant to Canada, No. (%)	244 (22.8)

Injection drug use, No. (%)	Any injection drug use	No injection drug use
Immigrant to Canada	24 (4.2)	158 (44.1)
Born in Canada	551 (95.8)	200 (55.9)
Unknown immigration status	40	4
Have any children, No. (%)	810 (73.0)	
Housing insecurity, No. (%)	100 (10.6)	
University or college-level education, No. (%)	364 (38.6)	
Employed, No. (%)	324 (29.7)	
Incarceration history, No. (%)	294 (28.1)	
Current alcohol use, No. (%)	380 (35.0)	
>3 alcoholic drinks per day (high risk), No. (%)	41 (3.9)	
History of drug use, No. (%)	706 (70.6)	
History of IV drug use, No. (%)	615 (62.9)	
On OAT, No. (%)	260 (25.0)	
Diabetic, No. (%)	70 (6.7)	
Psychiatric diagnosis, No. (%)	389 (36.5)	
positive, No. (%)	52 (5.6)	
HBsAg positive, No. (%)	18 (2.2)	
HCV treatment naïve, No. (%)	1053 (90.2)	
Reinfected, No. (%)	56 (5.4)	

HCV Genotype by Country of Origin, No. (%)	Genotype 1	Genotype 2	Genotype 3	Genotype 4	Genotype 5	Genotype 6	Multiple Genotypes
Africa	1 (0.2)	1 (1.1)	1 (0.4)	16 (76.2)	0 (0)	0 (0)	1 (11.1)
Asia	40 (7.5)	12 (13.6)	31 (12.9)	2 (9.5)	0 (0)	9 (100)	3 (33.3)
Europe	50 (9.3)	6 (6.8)	4 (1.7)	1 (4.8)	0 (0)	0 (0)	0 (0)
North America	441 (82.1)	67 (76.1)	201 (83.8)	2 (9.5)	1 (100)	0 (0)	5 (55.6)
Oceania	0 (0)	1 (1.1)	1 (0.4)	0 (0)	0 (0)	0 (0)	0 (0)
South America	5 (0.9)	1 (1.1)	2 (0.8)	0 (0)	0 (0)	0 (0)	0 (0)
Missing	537	88	240	21	1	9	9
Fibrosis score, No. (%)							
F0-F1			706 (66.8)				
F2			81 (7.7)				
F3			97 (9.2)				
F4			173 (16.4)				
Baseline FIB-4 value, mean (SD)			1.93 (2.86)				
Cirrhotic, No. (%)			173 (15.0)				
Initiated DAA treatment, No. (%)			1053 (90.2)				
Cis women			1031 (90.3)				
Trans women			14 (82.4)				
Trans men			8 (100)				
Achieved SVR, No. (%)			785 (99.0)				

Abbreviations: CANUHC, Canadian Network Undertaking Against Hepatitis C; DAA, direct-acting antiviral; FIB-4, Fibrosis-4 Index; HBsAg, hepatitis B surface antigen; HCV, hepatitis C virus; IV, intravenous; OAT, opioid agonist therapy; SVR, sustained virologic response.

Cirrhotic patients were more likely to be newcomers and immigrants (30.3% vs 21.6%) and unemployed (79.9% vs 69.0%) than noncirrhotic patients. Cirrhotic patients were less likely to have a history of recreational drug use (62.6% vs 71.7%), to have a psychiatric diagnosis (23.5% vs 38.4%), and to receive opioid agonist therapy (OAT; 16.8% vs 26.2%). Reinfected patients were more likely to have a history of recreational drug use (87.2% vs 69.5%), to have a psychiatric diagnosis (55.6% vs 34.4%), to receive OAT (39.0% vs 24.3%), to have a history of incarceration (47.6% vs 26.8%), and to be HIV seropositive (15.2% vs 5.6%).

Most participants (90.2%) were treatment naïve before their index assessment, and 5.4% were reinfected. The most common HCV genotype in the cohort was 1a (45.5%). The mean AST to Platelet Ratio Index (APRI) was 0.8 (1.1), mean Fibrosis-4 (FIB-4) Index for Liver Fibrosis was 1.93 (2.86), and the fibrosis stage for the majority was F0-F1 (66.8%). Fifteen percent were cirrhotic, and of those, 13.3% had decompensated liver disease (Table 1).

**Figure 1.**
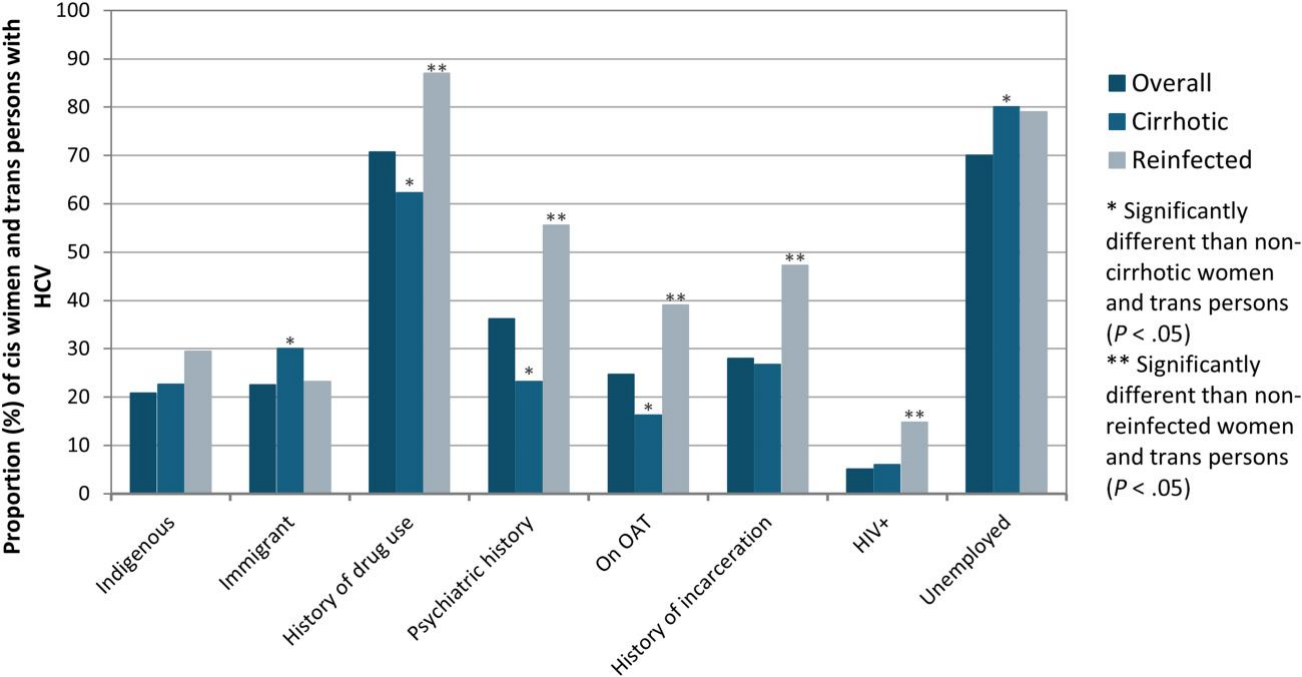
Proportion of those with cirrhosis and reinfection.

A total of 1053 (90.2%) participants initiated DAA treatment. Of the remaining individuals, 8 individuals (7.0%) were previously treated and cured, 16 (14.0%) were yet to start therapy, 11 (9.6%) spontaneously cleared infection, and 79 (69.3%) were missing DAA treatment information. SVR follow-up data were available for 793 of 1053 (75.3%) HCV patients. Treatment uptake was similar among trans persons (14/17 vs 8/8) as among the total cohort. SVR was achieved by 785 of 793 (99.0%) patients who completed therapy and had available post-treatment HCV RNA testing information and by 74.6% of 1053 patients who initiated DAA treatment. Of the 260 individuals who did not have RNA testing at SVR12, 3 (1.2%) had a testing date but no result entered, 3 (1.2%) had died, 19 (7.3%) were not able to be contacted, and 10 (3.9%) had a treatment interruption. Reasons for lack of testing were not present for the other 225 individuals. Those with missing RNA results at SVR12 were younger (47.9 vs 50.2 years; *P* = .02) and more likely to be white (72.5% vs 58.6%; *P* = .003), to be injection drug users (70.8% vs 59.9%; *P* = .004), and to experience housing insecurity (15.1% vs 9.2%; *P* = .02) and were less likely to have earned a university or college degree (29.7% vs 42.9%; *P* = .0008) than individuals who completed RNA testing (Supplementary Table 1). Comparisons of cirrhotic with noncirrhotic and reinfected with nonreinfected cis women and trans persons are reported in Figure 1.

## DISCUSSION

This study utilized data collected in 17 specialty centers across Canada to develop a large cohort of >1000 cis women as well as 17 individuals who identified as trans women and 8 who identified as trans men. While overall there were few trans persons in the cohort, these data represent a larger cohort of individuals with HCV who identify as trans, and therefore adds to the literature in the Canadian context; as this is a gap often identified by public health, policy, and decision-makers and experts in HCV care and treatment.

In our study, we found that the highest risk for acquisition based on past experiences was recreational drug use and that a high proportion had a history of experience in the correctional system. The rate of reinfection in the cohort was 5.4%, which could be influenced by follow-up time. This reinfection rate is like what has been seen in other studies, and interestingly 1 study showed that reinfection is lower among women than men [7].

Most women in our cohort had not been involved in higher-risk sexual activities. A recent literature review using Socioecological Model and Theory of Gender and Power found that personal, interpersonal, community, and societal risks play a role in women acquiring HCV. Specifically, transitioning to drug use as a coping strategy without the knowledge of HCV contributed to personal risk, and housing instability was identified as a community risk [8]. Stigma has also emerged as a major theme affecting women seeking HCV care [9]. In a study of 296 women who used drugs in the United States, stigma was linked to decreased odds of liver disease staging and starting treatment. In contrast, strong social networks were linked to knowing a cure for HCV was available and to seeking HCV-related care in the prior 12 months [10]. In our cohort, strikingly, many who had used injection drugs were not on OAT. A better understanding of why this was the case should be the focus of future work (eg, interest vs engagement vs access). Furthermore, models of care that specifically focus on women in residential or outpatient drug treatment have demonstrated high HCV treatment initiation and completion [11]. Interestingly, these models have been linked to supporting the creation of a social network and, importantly, women re-claiming their role within the family to support recovery [12].

It has previously been shown that among trans women, even in the absence of injection drug use or incarceration, HCV seropositivity is quite high, and in 1 study it was 10% [3]. Treatment uptake in this same cohort of trans women was also high at 82%, although less than half completed SVR testing [3]. Interestingly, in multiple studies, over half of trans individuals already knew their diagnosis, demonstrating the need for linking or re-linking to care [3, 13].

Our data were collected in specialty centers, and therefore rates of advanced liver disease are higher than in the community. In our study, newcomers and immigrants were overrepresented in those with advanced liver disease. As HCV is not a part of the Canadian Immigration Medical Exam, and individuals who have immigrated from endemic countries may have acquired HCV vertically or as children, this represents a major gap in care. In fact, 1 Canadian study demonstrated that on average time from immigration to first HCV assessment was 17 years [14]. This cohort of women may face additional challenges to screening and linkage to care such as a lack of symptoms, stigma, experiences of ethnocultural discrimination, and a lack of access to primary care [15].

Finally, we noted that despite the overrepresentation of advanced liver disease, the mean APRI score was low and there was little hazardous alcohol use. However, it should be noted that alcohol use history was not in the context of lifetime, or even weekly use, and thus should be interpreted with caution. Of those with cirrhosis, the mean age was 10.4 years older than those without cirrhosis. Taken together, these data may support the use of history such as age and alcohol use to determine the likelihood of advanced liver disease and support rapid treatment starts.

### Limitations

This cohort relied partially on self-report and therefore is not verifiable. The proportion of DAA treatment recipients without post-treatment HCV RNA testing results was 24.7%, and we lacked details regarding the outcomes of these individuals; therefore, the high reported SVR may not reflect the entire cohort.

## CONCLUSIONS

Prioritizing disaggregated data and outcomes has been cited as a priority in the efforts toward the elimination of HCV as a public health threat and should be done regionally. This study describes a large Canadian cohort of cis women and a smaller cohort of trans persons. In the majority, fibrosis scores were low, and alcohol use was not excessive, which enables rapid treatment starts in the absence of a comprehensive assessment. From a harm reduction perspective, OAT uptake was suboptimal among those who use drugs, an observation that may support co-localizing HCV treatment with OAT initiation to prevent drug use relapse and reinfection. Finally, our data demonstrate that late-stage presentation and reinfection disproportionately affect certain communities and that additional novel efforts are required to address this issue.

## Supplementary Material

ofag356_Supplementary_Data

## Supplementary Data

Supplementary materials are available at *Open Forum Infectious Diseases* online. Consisting of data provided by the authors to benefit the reader, the posted materials are not copyedited and are the sole responsibility of the authors, so questions or comments should be addressed to the corresponding author.
