# Supplementary material for: A Gender-Based Descriptive Analysis of the Canadian Network Undertaking Against Hepatitis C (CANUHC) Cohort From 2015–2023
Source: Open Forum Infect Dis. 2026 Jun 6;13(6):ofag356. doi: 10.1093/ofid/ofag356 (PMC13293119; doi:10.1093/ofid/ofag356)
Supplement: ofag356_Supplementary_Data [file ofag356_supplementary_data.docx]

**Supplemental Table 1.** Comparison of baseline demographics according to lost to follow-up status among patients who initiated DAA treatment.

| **Characteristic** | | **SVR testing completed (n= 793)** | **Lost to follow-up prior to SVR testing (n=260)** | **P** |
| --- | --- | --- | --- | --- |
| Age in years, mean (SD) | | 50.2 (13.5) | 47.9 (14.4) | 0.02 |
| Missing | | 3 | 4 |  |
| Gender (self-identified), n (%) | |  |  |  |
|  | Cis Women | 777 (98.0) | 254 (97.7) | 0.92 |
|  | Trans Women | 10 (1.3) | 4 (1.5) |  |
|  | Trans Men | 6 (0.8) | 2 (0.8) |  |
| Race/ethnicity (self-identified), n (%) | |  |  |  |
|  | Asian | 83 (10.6) | 18 (7.2) | 0.003 |
|  | Black | 21 (2.7) | 6 (2.4) |  |
|  | Indigenous | 190 (24.3) | 37 (14.7) |  |
|  | White | 458 (58.6) | 182 (72.5) |  |
|  | Other | 30 (3.8) | 8 (3.2) |  |
| Immigrant to Canada, n (%) | | 195 (27.1) | 33 (13.5) | <0.0001 |
| Missing | | 74 | 16 |  |
| Injection drug use, n (%) | | 390 (59.9) | 160 (70.8) | 0.004 |
| Missing | | 142 | 34 |  |
| Have any children, n (%) | | 549 (72.9) | 181 (73.6) | 0.84 |
| Missing | | 40 | 14 |  |
| Housing Insecurity, n (%) | | 57 (9.2) | 33 (15.1) | 0.02 |
| Missing | | 173 | 42 |  |
| University or college level education, n (%) | | 276 (42.9) | 60 (29.7) | 0.0008 |
| Missing | | 150 | 58 |  |
| Employed, n (%) | | 236 (31.8) | 59 (24.7) | 0.04 |
| Missing | | 50 | 21 |  |
| Incarceration history, n (%) | | 191 (26.9) | 69 (29.7) | 0.41 |
| Missing | | 84 | 28 |  |
| >3 alcoholic drinks per day (high risk), n (%) | | 34 (4.8) | 6 (2.5) | 0.06 |
| Missing | | 87 | 24 |  |
| History of drug use, n (%) | | 453 (67.7) | 177 (77.0) | 0.008 |
| Missing | | 124 | 30 |  |
| History of IV drug use, n (%) | | 390 (59.9) | 160 (70.8) | 0.004 |
| Missing | | 142 | 34 |  |
| Psychiatric diagnosis, n (%) | | 247 (34.5) | 87 (35.2) | 0.83 |
| Missing | | 76 | 13 |  |
